# Moving robotics competitions virtual: The case study of RoboCupJunior Soccer Simulation (SoccerSim)

**DOI:** 10.3389/frobt.2022.915322

**Published:** 2022-08-15

**Authors:** Felipe N. Martins, Adrián Matejov, Marek Šuppa

**Affiliations:** ^1^ Sensors and Smart Systems Group—Research Centre Biobased Economy/Institute of Engineering, Hanze University of Applied Sciences, Groningen, Netherlands; ^2^ Department of Applied Informatics, Comenius University, Bratislava, Slovakia

**Keywords:** educational robotics, robotics competitions, RoboCup, RoboCupJunior soccer, robot simulation, robot soccer, SoccerSim, COVID-19 pandemic

## Abstract

For almost 25 years, the goal of the RoboCup has been to build soccer robots capable of winning against the FIFA World Champion of 2050. To foster the participation of the next generation of roboticists, the RoboCupJunior competition takes place in parallel and provides a similar challenge of appropriate difficulty for high school students. RoboCupJunior has three main categories: Soccer, Rescue and OnStage. For the Soccer category, participants need to design, build and program a team of autonomous robots to play soccer against an opponent team of robots. The competition is physical in nature, since it assumes physical robots playing against one another. In 2020 and 2021, the COVID-19 pandemic has made it difficult for a competition of this type to take place, due to obvious restrictions on physical gatherings. To allow for some sort of participation, and inspired by positive experience of the larger RoboCup community, the Organizing Committee of RoboCupJunior Soccer has explored porting a portion of the challenge to a simulated environment. Many of the existing environments, however, are built for higher education/research teams competitions or research, making them complex to deploy and generally unsuitable for high school students. In this paper we present the development of SoccerSim, a simulated environment for RoboCupJunior Soccer, based on the Webots open-source robotics simulator. We also discuss how the participation of students was key for its development and present a summary of the competition rules. We further describe the case study of utilizing SoccerSim first as a testbed for a Demo competition, and later as part of RoboCup Worldwide 2021. The participation of more than 60 teams from over 20 countries suggests that SoccerSim provides an affordable alternative to physical robotics platforms, while being stable enough to support a diverse userbase. The experience of using SoccerSim at RoboCupJunior Worldwide 2021 suggests that a simulated environment significantly lowers the barrier to entry, as evidenced by the participation of many teams that have not participated before. To make it easy for similar competitions to take place in the future, we made the code of SoccerSim available as open-source, as well as the associated tooling required for using it in a tournament.

## 1 Introduction

The use of robots as educational tools has been shown to provide a stimulating learning environment, promoting active learning with long-lasting impact (Ahlgren, 2002; Alves et al., 2011; Aroca et al., 2013). In their systematic review of studies on educational robotics, Anwar et al. (2019) concluded that educational robotics “has potential as a learning and teaching tool, including supporting the education of students who do not display immediate interest in academic disciplines related to science or technology”. This has also been emphasised by Eguchi, (2016), who has shown that students that participated in the robotics competitions of RoboCupJunior reported enhanced interest and learning of STEM contents, computational thinking and engineering skills. According to Brancalião et al. (2022), many robotics competitions contribute to education by increasing students’ interest in STEM concepts, connecting students to industry professionals, and enabling them to solve real world problems. Finally, Aroca et al. (2016) point out that robotics competitions can also have a positive social impact because they motivate integration between schools, universities and the larger community.

RoboCup is an international robotics competition organized annually since 1997 with the aim of promoting science and engineering research in robotics and artificial intelligence. Its *dream* is: “By the middle of the 21st century, a team of fully autonomous humanoid robot soccer players shall win a soccer game, complying with the official rules of FIFA, against the winner of the most recent World Cup” (RoboCup Federation, 2022). Since its first edition, RoboCup evolved beyond soccer competition, as Ferrein and Steinbauer (2016) detail in their article “20 Years of RoboCup”. Currently, the competition is divided into five leagues: Soccer, Rescue, @Home, Industrial and Junior, with a total annual count of between two and three thousand participants from more than 40 countries. The Junior league focuses on educational robotics and targets students between 14 and 19 years old. It is sub-divided into Soccer, Rescue and OnStage leagues, and typically counts about a hundred teams of students from more than 30 countries all around the world.

Robot soccer simulation is part of the RoboCup competition since its first edition in 1997 (Kitano et al., 1997). Because there is no need to build and maintain hardware, the main focus of simulation competitions is on artificial intelligence and team strategy. What was initially a top-view 2D simulation of robot soccer evolved into several simulation categories in the leagues Soccer 2D and 3D, Rescue Agent Simulation and Rescue Robot (RoboCup Federation, 2022). The Junior league of RoboCup was inaugurated in the year 2000, but without any simulation-based categories. Simulation was introduced in Junior league only in 2013 with CoSpace robotics (Eguchi and Shen, 2013), which combined simulation with real robots in a rescue competition. More recently, CoSpace was replaced by Junior Rescue Simulation^1^.

A demonstration of Soccer Simulation was first introduced in RoboCupJunior only in February 2021. But there are earlier examples of soccer simulators for school-aged children proposed by different groups, which illustrates the interest in this kind of tool. For instance, years ago the authors of this study were independently working on two initiatives, almost simultaneously, but without knowing about each other. Šuppa and Matejov, (2014) released py-soccersim at the end of 2014, which is a 2D robot soccer simulator built in Python 2. By its turn (Martins et al., 2015; Martins et al., 2016), were working on a very similar project: RoSoS, a Robot Soccer Simulator based on Processing^2^ to be used in robotics competitions by primary and secondary students. We also proposed a Junior Soccer Simulation League because many students may not be able to participate in robotics competitions due to financial limitations, especially in developing countries. In many cases, neither the students nor their school can afford the costs of robotics kits and related infrastructure in order to prepare for the competitions. In such context, an open-source simulator could help reduce the barrier for students to get involved in educational robotics and robotics competitions, which contributes to education equality.

Both py-soccersim and RoSoS were designed to be simple. But, besides being open-source, neither initiative was widely adopted, perhaps because setting up the simulation required some programming knowledge and/or because a 2D-based simulation was not very appealing to students. More recent projects were independently developed by organizers of competitions in Australia and Iran, and had been used recently in their respective countries. RCJA SoccerSim was developed by RoboCupJunior Australia (2021) and implements a soccer simulator that requires no installation nor configuration because it works directly in the web browser. The simulator has both Blocks and JavaScript-based editors, which means it can be used even by students with very little programming experience. The disadvantage is that RCJA SoccerSim is still a top-view 2D-based simulation, like py-soccersim and RoSoS. On the other hand, the Iranian Junior Cup Competition Committee, (2022) developed a realistic 3D Soccer Robot Simulator that mimics the real RCJ soccer field. Their virtual robots can be programmed in Python or C++ and are very similar to the ones used in real competitions, featuring many sensors and a 4-wheel omnidirectional drive. The Junior Cup simulator is very powerful and visually appealing, but it lacks a little on integration because the simulator and the robot code run in separate instances. Also, the 4-wheel omnidirectional robot is more complex to control, which increases the difficulty for beginners.

In this paper we present the development of SoccerSim for RoboCupJunior Soccer, which is a 3D simulator designed to be both visually appealing and very easy to setup. SoccerSim is based on Webots (Cyberbotics Ltd., 2022), a professional open-source robotics simulator responsible for the computation of the simulated physics and the interaction between the robots and the environment. The text of the paper is organized as follows: In Section 2 we explain the general approach of this work, the robotics simulator used to build SoccerSim and the rules of the competition. In Section 3 we describe SoccerSim, and present the case study of utilizing it as part of the RoboCupJunior, including how the participation of students was key for the development of the software and the competition. Finally, a discussion is presented in Section 4, followed by an outline of planned future work.

## 2 Materials and methods

In the years 2020 and 2021, the global pandemic of COVID-19 has made it difficult (if not impossible) for a competition like RoboCupJunior to take place due to the restrictions on physical gatherings. To allow for at least some participation of students, the Organizing Committee was faced with the following question:

How can RoboCupJunior Soccer competition be implemented in a virtual environment, in which teams are physically separated?

In 2020, there was not enough time to organize a full virtual competition, so the Organizing Committee decided to host only a virtual poster session for teams to present the robots that they had developed. For 2021, however, the committee decided that a virtual competition would take place. The main decisions were:

1. Host the Junior event in GatherTown^3^, an on-line platform that provides an immersive experience for team interactions. An environment was created to host all meetings, presentations and talks between students and the organizers.
2. Design some challenges for students to work on at home/school. Each team would have some limited amount of time to solve a challenge using their own physical robots at home.
3. Host a Soccer Simulation competition. This also meant that the software for simulating the soccer matches needed to be developed.

The regular RoboCupJunior Soccer competition requires students to build their own robots, and teams usually start working several months (up to a year) prior to the event. Therefore, by the time the Organizing Committee decided to turn the event into a virtual competition, many teams were already working on their robots, and some had their robots already working. Considering that fact, the Organizing Committee decided to create the challenges mentioned in item 2 to recognize and value their effort. It is important to point-out that teams could choose to participate either in the physical challenges (item 2), or in the simulation competition (item 3), or both.

In this paper we focus on item 3 to present the case study of RoboCupJunior Soccer Simulation. The process was divided in the following tasks:

• Development of a beta version of SoccerSim software.
• Run a demo competition in February 2021 to assess the software and rules.
• Make necessary adjustments in the software and rules based on results and feedback from participants.
• Develop the final version of SoccerSim.
• Run the official competition in June 2021.

For the development of the SoccerSim software, a few criteria were defined: the software needed to be open-source, its development needed to be open for students contribution, and it should allow students to program in Python (as Python is growing in terms of adoption and popularity in the recent years^4^). The competition rules were developed by the RoboCupJunior Soccer Technical Committee, in collaboration with the developers of SoccerSim. A few threads were started in the RoboCupJunior Forum^5^ to get suggestions and feedback from the students and the community.

In December of 2020 we started organizing the first demo competition to assess software performance and rules. A call for participation was posted in the RCJ Forum^6^ and the competition happened in February 2021. With the feedback collected from participants, the necessary adjustments in the SoccerSim software and rules was made, and the Worldwide competition took place in June 2021.

During the entire process we collected qualitative feedback from students and mentors *via* RCJ Forum. We also sent out a survey after the competition to assess how students liked it and to collect suggestions for improvement.

In the following subsections we describe Webots, the simulator used to build SoccerSim, and explain the competition rules.

### 2.1 Webots robot simulator

Webots is an open-source robotics simulator software developed and maintained by Cyberbotics Ltd. (2022) since 1998 (Michel, 2004). It is an integrated development environment (IDE) capable of running on Linux, MacOS as well as Windows. The environment itself contains tools to build a world with objects of different types from solid ones to the robots with sensors, motors and wheels. It contains an asset library with many robots, sensors, actuators, objects and materials, which can be used when building your own simulated world. Every robot created in the environment can be controlled by a custom user-provided program. The user may choose between different programming languages, ranging from interpreted languages like Python and/or Java to compiled ones such as C and/or C++. Moreover, it is possible to designate a specific program to be a so called supervisor, which is capable of accessing and manipulating other objects in the simulation.

As Hughes et al. (2019) suggest, Webots is well suited for young students due to its compatibility with multiple operating systems (including Windows) and its use as educational tool. It can also be called self-contained in the sense that its installation contains everything that is necessary for a student to start experimenting with their own robot programs. At the same time, it does not limit the use of well established software engineering practices by advanced students, such as object-oriented decomposition and source code versioning, which are the prerequisites for collaborative software development, the experience of which RoboCupJunior aims to foster among the participating students. Moreover, Webots was also used for the implementation of EREBUS, a simulation competition environment for the RoboCupJunior Rescue competition (RoboCupJunior Rescue Committee, 2021). For those reasons, Webots was chosen as platform for the implementation of SoccerSim for the RoboCupJunior Soccer. We used Webots version R2021a in the Demo competition, and version R2021b in the RoboCup Worldwide 2021.

### 2.2 Competition rules

A single game consists out of two teams playing against each other for 20 min (two halves of 10 min each, one on each side of the field). Each team consists of three programmable robots, which try to move the ball into the opponent’s goal. Similarly to a real world soccer game, the team with the higher number of scored goals at the end of the 20 min wins. A detailed description of SoccerSim rules can be found in (RoboCupJunior Soccer Technical Committee, 2021). In SoccerSim, similarly to other RoboCupJunior challenges, programming has to be performed exclusively by the students. The same code is used during the whole competition and/or tournament.

The regular physical competition uses two robots per team because it is the minimum number to form an actual *team*, and it requires students to implement at least a very basic collaboration strategy. Increasing the number of robots per team is desirable, but could result in prohibitive increase in costs, limiting the participation of schools and students. Such cost limitation does not exist in simulation, so we decided to use three simulated robots per team in SoccerSim. It does result in an increased complexity in terms of programming and strategy, but it is somewhat compensated by the lack of hardware issues.

**FIGURE 1:**
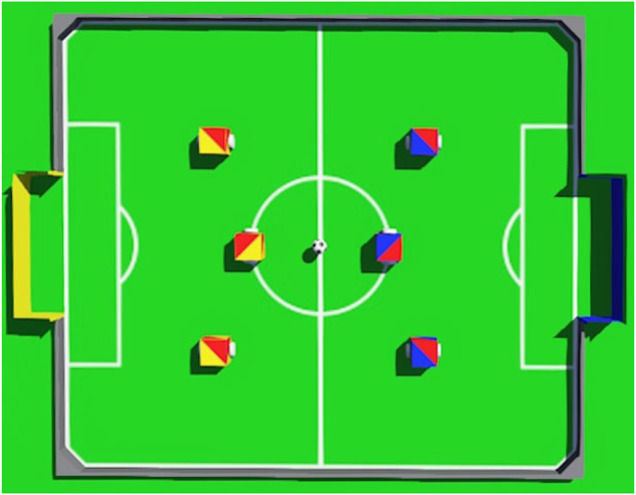
Top view of the simulated soccer field with the robots and the ball positioned on the seven neutral spots.

The simulated robots make use of differential-drive, which means that they have two independently controlled wheels. The robot program can only change the speed of each wheel to control the robot’s movement. The robots must use the ball to score into a color-coded goal on a special field that resembles a human soccer field. The field contains seven so-called *neutral spots*, which are located in the positions occupied by the robots and the ball in Figure 1.

As much as possible, SoccerSim rules are the same as (or very similar to) the rules of the physical RoboCupJunior Soccer category (RoboCupJunior Soccer Technical Committee, 2022). To suit the simulated robot scenario, however, some rules had to be adapted. Perhaps the most significant change concerns refereeing: in SoccerSim, the automatic referee makes all the decisions during the game, including the placement of the ball and of robots. Furthermore, there is a situation when the ball is stuck between robots or is beyond detection or reach capability of all robots on the field. In such cases, the referee (in our case supervisor) calls *Lack of progress* and moves the ball to the *nearest* unoccupied neutral spot. The same rule applies to robots that do not make sufficient progress, i.e., do not travel a certain distance for 15 s. This prevents blocking of the ball, of other robots and helps ensure the robots are “reset” from situations in which their movement might get restricted indefinitely (i.e., when they accidentally turn upside down).

Another SoccerSim-specific rule is preventing robots from staying inside their penalty area for too long, so a team cannot block the entire goal and do nothing else. If any of the robots stays in the penalty area for more than 15 s, the supervisor moves the robot to the *farthest* unoccupied neutral spot from its current position.

Table 1 presents a summary of the main differences between the physical and the simulated competitions.

**TABLE 1:** Summary of the main differences between the physical and simulation competitions of RoboCupJunior Soccer.

Aspect	Physical competition	Simulation competition
Robots per team	Two	Three
Robot features	Built by the students. Size and weight is limited per category: 18.0 (or 22.0) cm in diameter and height, 2.2 (or 1.1) kg in weight. Number and format of wheels is not regulated. Can have a ball kicker	All robots are cubic-shaped (8 × 8 × 8) cm, differential-drive with two wheels. Only the speeds of the wheels can be controlled, but are limited in simulation. Robots do not have a ball kicker
Sensors	Any number and type of sensors is allowed, as long as it does not disturb other robots	Currently available sensors are: GPS, compass, 4 sonars (one on each side), and ball IR sensor
Ball	Depends on the category: IR-emitting ball (Lightweight) or orange golf ball (Open)	Simulates an IR-emitting ball
Playing field	(1.93 × 1.32) m, carpet floor, marked by white lines and surrounded by walls distant 25 cm from the lines. Robots are not allowed to leave the playing field	(1.50 × 1.30) m, flat surface, surrounded by walls that keep the robots and the ball inside the playing field
Referees	Two human referees (the main referee and an assistant) make decisions and enforce the competition rules	Competition rules are enforced by the automatic (software) referee running during the simulated match. No human interference is allowed or required

## 3 Results

In this section we present a description of the developed soccer simulator and describe some of its features. We also show the results of using SoccerSim to implement a soccer simulation competition at RoboCup Worlwide 2021, including how students participated in the development process, and how their feedback helped shape the resulting software.

### 3.1 SoccerSim

**FIGURE 2:**
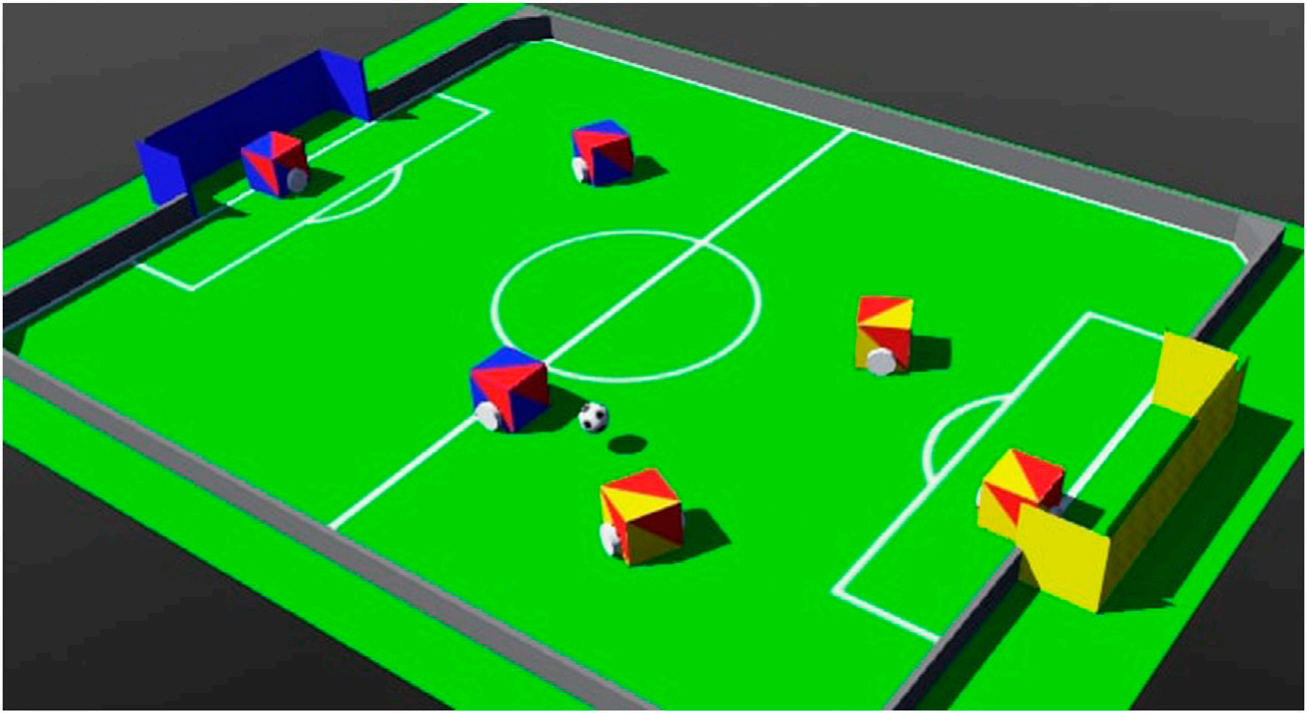
SoccerSim screenshot: Six cubic-shaped robots and a ball are shown inside the soccer field, which is surrounded by a wall. The goals are painted in yellow and blue, like in the physical RoboCupJunior soccer fields.

The RoboCupJunior Soccer Simulator (SoccerSim) was based on the demo world *soccer.wbt*, which is shipped as part of Webots. This demo world simulates an environment in which six cubic-shaped differential-drive robots (three per team) can be programmed individually to play soccer (Figure 2). Our contribution was the adaptation of this environment to the rules and conditions of the RoboCupJunior Soccer. The main one was the creation of a supervisor program that implements the Soccer Simulated rules summarized in Section 2.2, acting as an automatic referee. The supervisor also displays messages during the match, highlighting relevant events according to the SoccerSim rules. Other modified components included the ball (to emit infrared), and the robots (to be able to use more sensors, like compass, sonar and the IR-ball sensor). Dedicated supervisors and environments were also created for the technical challenges explained in Section 3.4.5. Finally, a video recorder module was also implemented. More information is provided in the following paragraphs, and details are available at the GitHub repository of SoccerSim^7^.

Controlling robot objects in Webots is performed by a specific program called *controller*. The user must define the name of the controller that a particular robot should be controlled by. These controllers are executed on startup of the simulation in separate processes. Although Webots supports multiple programming languages, we decided to primarily provide support for the Python programming language mainly because of its clear and intuitive syntax that allows students to quickly express their solutions in code (Fangohr, 2004), so even students without a significant background in programming could join the competition with ease. The program to control the robots can also make use of Python’s builtin libraries, as well as *numpy* (Harris et al., 2020) and *scipy* (Virtanen et al., 2020), which are considered the standard Python libraries for scientific computing.

Within a Webots world, it is also possible to create so called supervisor robot objects, which are able to manipulate other objects in the simulated world they exist in. In fact, we created such a supervisor in SoccerSim and used it as a referee. It is implemented by a Python 3 script as well. For simplicity and readability, the referee’s code is split into several files, utilizing an Object Oriented Programming (OOP) paradigm, with clear separation of concerns. Since the referee is also the supervisor, it makes it easy to check whether robots follow the rules or a goal has been scored. The position of each robot is tracked while periodically checking whether rule-offending events like lack-of-progress or inside-penalty-area took place.

The source code contains sample robot controllers for two teams playing against each other to provide a starting point for potentially interested students. Each robot is equipped with sensors (signal emitter and receiver) and two motors (summarily referred to as devices), that the participants are allowed to make use of. The main task for the robot is to find and seek the ball on the field. In earlier versions of SoccerSim, the supervisor distributed this information packaged into messages *via* a communication channel to every robot in the field: The positions of the ball and robots was communicated to all robots. In the current SoccerSim version, the robots must use their own sensors to detect the ball and the other robots. To make the scenario a bit more realistic, the measurements from the onboard sensors include a small amount of noise, which the participants need take into account. For example, when the robot is very next to an obstacle, the value returned by the sonar sensor is 0, with an uncertainty of 0%. On the other hand, when the robot is very far and the sensor does not detect anything, the value returned is 1,000 with an uncertainty of 5%. The sensor values and associated uncertainty are linearly interpolated.

The movement of the robot is actuated by setting a velocity for each of the two motors and is expressed in radians per second.

Important part of the physical robots is information exchange, which makes it possible to collaborate on a specific task. There is a wide variety of devices supporting the communication to choose from (Bluetooth, WiFi, radio etc.). Since Webots supports Emitter and Receiver devices, we decided to mount both of those to each robot. Both teams communicate on different channels so it is not possible to intercept or interfere with foreign messages. When a message is sent to a channel, it is distributed to all of the teammates listening on that channel. Communication in soccer might be useful when it comes to splitting the roles of the robots or changing the strategy entirely on a dynamic basis.

Webots also provides an option to record a video from a specific simulation. We implemented a video recorder assistant submodule for that matter, which supports creating either MP4 or HTML5 output formats, by making use of the Webots video API. At the same time, the architectural design of the supervisor allows for various “game events” to be emitted, such as

• MATCH_START, which denotes the beginning of the simulated match
• MATCH_FINISH, which denotes the end of the simulated match
• LACK_OF_PROGRESS, as described in Section 2.2
• INSIDE_PENALTY_FOR_TOO_LONG, as described in Section 2.2
• KICKOFF
• GOAL

**FIGURE 3:**
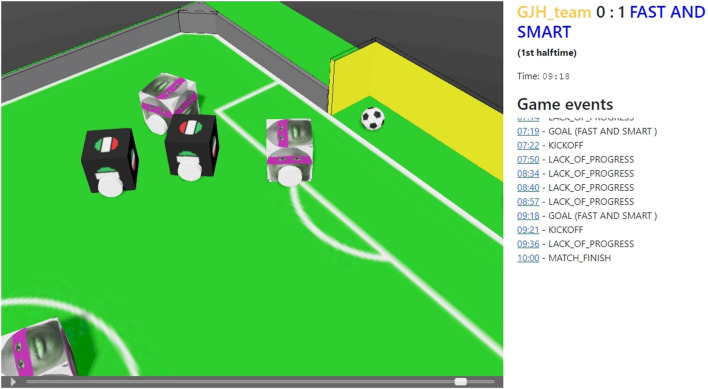
Visualization tool based on the HTML5 output of a match between GJH team and FAST AND SMART. A player for the match is shown in the left side, which allows for play, pause, fast forward, rewind, zoom and change view controls. In the right side the game events and *clickable* associated time-stamps are shown.

Each of these is associated with the “real world” timestamp as well as the match time at which it took place. Additionally, each of them can have a custom payload attached, for instance the resulting score when a goal is scored or the name of the offending robot that was inside the penalty area for too long. The events are processed through a central handling system, which also allows for custom handlers to be added. These can then consume the events and react to them in various ways, for instance by serializing them into a JSON Lines^8^ file called “reflog” (an abbreviation for “referee” and “log”) which in the end contains the log of all the actions the referee took, or rendering them onto the screen. This architectural design would then make it easy to make further use of these events, for instance by sending them to third-party systems for further processing. Additionally, the existence of these “events” then allows for an enriched HTML5 video playing experience, which also contains links to the game times at which a respective event took place, thanks to which one can “jump” to any specific game event of interest (Figure 3).

SoccerSim software supports specifying most of the settings of its game-simulating environment, like game time, team names, initial scores, output format of the video and so on. In addition to running from within the aforementioned IDE, SoccerSim can also be executed in a “headless” mode in a Docker container, the basis of which is provided by the developers of Webots themselves. The resulting Docker container, which combines the SoccerSim software with the Webots base, can then be run on essentially any Unix-based computer, making the simulation of SoccerSim-powered matches as easy as running a specific command, while also being easily parallelizable across multiple machines.

Full source code of the referee (supervisor), sample robots and definition of Webots world is located at GitHub^9^. Instructions on how to set up the environment can be found in the README file or in the documentation (Matejov et al., 2021).

### 3.2 SoccerSim demo competition

As described in Section 1, SoccerSim has first been introduced as a “demonstration challenge” during the RoboCup Worldwide 2021. When discussing its potential inclusion, however, it transpired that using it directly in an international competition without extensive prior testing might prove too dangerous. In order to provide an avenue for such testing, the “SoccerSim Demo Competition 2021” has been organized roughly 6 months in advance. Its aim was to give the potential competitors an early access to the SoccerSim software, evaluate the use of Webots as a platform for robotic soccer simulation, its appropriateness for high school students, and gather feedback that would later be incorporated in an updated version prepared for RoboCup Worldwide 2021. The Demo competition has been announced on RoboCupJunior Soccer forum^10^, as well as *via* the regional representatives of RoboCupJunior across the world and attracted considerable attention: in the end, over 50 teams from 18 countries have registered their interest to participate, with 41 of them actually submitting their robot’s programs. We hypothesize that this significant interest was caused by the novelty of the challenge (the first time a simulated challenge associated with RoboCupJunior Soccer took place) as well as the fact that it took place at the height of the COVID-19 pandemic (late 2020, early 2021).

Predictably, the experience has yielded a lot of valuable feedback. Perhaps the most significant lesson learned was the fact that simply posting submission instructions and letting the participants upload their programs does not necessarily produce a package that can be directly used with the SoccerSim tournament simulation tooling. Out of 41 code submissions we received, not a single one was formatted in a way that would be directly consumable by SoccerSim’s automation, which forced the organizers to invest a non-trivial amount of time into manually fixing the submissions. In order to avoid this situation in the future, two approaches have been implemented. An automatic submission checker, which ensured the submission was formatted correctly, has been implemented in form of a simple static website that performed client-side validation of the submitted ZIP file. While this was certainly an improvement over the previous status quo, the correct structure of the submitted ZIP file did not guarantee that the submitted code would behave in the exact same way in the simulated environment just as it did when the students prepared it within the Webots IDE. In order to provide this guarantee, we built an open-source, Python-based web application called SoccerSim Checker^11^. It provides a way for participants of SoccerSim competitions to upload their code to a server which then automatically simulates a short, 10 s match against a sample team of opponents, which lets the competitors ensure that their submission does work similarly to what they observed when preparing it. As a byproduct, it provides a single place where all the submissions are located, which further simplifies the organization of the tournament.

Despite the organizational hiccups described above, the Demo competition has proven the Webots environment to be suitable for soccer simulation, especially when aimed at students within the RoboCupJunior target group (high school age). The controllers (e.g., the referee) prepared as part of the SoccerSim package were also shown to be rather stable and robust, with only a few minor fixes necessary for it to be used as part of the RoboCup Worldwide 2021.

### 3.3 Participation of students in the development of SoccerSim

The participation of students was key for the development of the entire RCJ Soccer Simulation competition, including the SoccerSim software. As explained in Section 2, we had a Demo competition in February 2021 to test the first version of SoccerSim and to collect feedback and suggestions from the participants. After the demo competition we created a topic in the RCJ Forum^12^ calling for participants to give feedback and suggestions.

Students were very active in the forum. A few examples of bugs that were identified by them are:

• SoccerSim was assigning the same code to two different robots of a certain team, but only when the team was in one of the sides of the field.
• In one situation, SoccerSim mistakenly considered a goal to be scored when the ball was pulled out of the field.
• The ball would move on its own when no robot was moving during the match.
• The match time was not in sync with the simulation time when the simulation was speed-up in Webots.

Let us emphasize that the above mentioned bugs were not identified by the development team before the demo competition. The active participation of the students was fundamental for the identification of the problems that were treated and corrected in the next updated version of SoccerSim that was used during the official competition in June 2021.

For the official competition we also made a call in the RCJ Forum asking for suggestions for the Technical Challenges^13^, and many different ideas were proposed.

### 3.4 SoccerSim challenge at RoboCup Worldwide 2021

As the introduction of Section 2 suggests, the RoboCup Worldwide 2021 competition necessarily needed to be different in many regards compared to previous international RoboCup competitions. Much of this stemmed from the inability of all competitors to be physically in one place at the same time. While this limitation has brought various organizational challenges, it also provided an opportunity to explore new ways of organizing an event that have not been tried before. We describe these in the subsections that follow.

#### 3.4.1 GatherTown

The RoboCup competitions are known for concentrating significant numbers of robots and robotists at the same venue, creating a very distinct atmosphere of a common set of goals (robots solving various challenges), serendipitous communication and technological innovation. While recreating such an atmosphere is very difficult to do in a virtual setup, we had a positive experience using the GatherTown platform to the same effect.

The GatherTown platform provides an immersive experience for meetings of multiple participants. In its environment, each participant is represented by its own avatar, making the whole environment look similar to the so called “role-playing games” (RPG). It is perhaps due to this familiarity, as well as to its playful nature, that the environment has been very well received by our target audience (mostly high-school students).

The RoboCupJunior Soccer GatherTown environment has been used as a common place for every participant to “exist in” during the 6 days when the competition took place. It was used for a wide range of activities, such as the competition-wide meetings, in which the organizers provided updates and responded to any questions, the poster session, in which the participants presented their work in form of a poster, interviews, in which the teams discussed their work with panel of judges, as well as spontaneous one-on-one discussions. By default, GatherTown only shares the video and audio output of a person’s avatar with other avatars in its vicinity, which tries to mimic the real-life experience of large gatherings.

#### 3.4.2 Daily gatherings

One of the most significant issues with virtual gathering that expect worldwide participation is the issue of timezones. While doing many activities asynchronously would be an option, we opted for synchronous release of news and updates, so that every participant would receive it at the same time while limiting the organizational overhead. As our participant’s location timezones ranged from the West Coast of US to the East Coast of Australia, we chose to meet at 15:00 UTC, which was deemed to be the least bad option.

To deliver the updates, the organizers used the “announce” feature, which propagates the audio and video stream of the announcer to everyone in the respective GatherTown. The announcements generally included the results and highlights from the individual and SuperTeam competitions, as well as the technical challenges, which we describe in greater detail in the sections below.

#### 3.4.3 SoccerSim individual competition

In the SoccerSim individual competition, the teams submitted their SoccerSim programs before the competition started. These were then used to simulate a whole tournament consisting of^14^

• Home round
• Away round
• Round of 16
• Quarter finals
• Semi finals
• Finals

For dramatic effect, the results of each of these rounds were released with daily cadence. For the Semi finals and Finals, commented live streams have been organized, which we describe in greater detail below. The results of all matches were then shared at the competition’s website, along with the recordings^15^.

#### 3.4.4 SoccerSim SuperTeam competition

The SuperTeam competition has a long tradition in the RoboCupJunior community. It consists of combining teams from different countries (and often times also continents) into one so called “SuperTeam”, which then solves the league’s challenge together, as a single (albeit virtual) team. In case of SoccerSim, with three robots per team, it was only natural to combine three teams into one SuperTeam. These newly formed teams were then tasked to collaborate on creating a single SoccerSim submission, which was then used to simulate the SuperTeam tournament, eligible for a different set of prizes than the SoccerSim Individual competition.

#### 3.4.5 Technical challenges

Inspired by the RoboCup major leagues and the need for further technological advancement of the leagues, technical challenges became a standard part of the RoboCupJunior Soccer international competition since 2018^16^. The idea of these challenges is to give the teams an opportunity to show off various abilities of their robots which may not get noticed during the regular games. Furthermore, the RoboCupJunior Technical Committee envisioned these challenges to be a place for testing new ideas that may make it to the future rules, or otherwise shape the competition.

We decided to adopt these challenges also into SoccerSim competition. Students had 24 h to reprogram their robots based on the description of the challenge. There were three challenges in total:

1. The fast goal shooter, to shoot as many goals into empty goal as possible within 2 min
2. Pass the ball, to pass the ball as many times as possible within 2 min
3. Precision shooter, to detect opponent’s robot using *Lidar* sensor and score as many goals as possible into the goal blocked by these robots.

**FIGURE 4:**
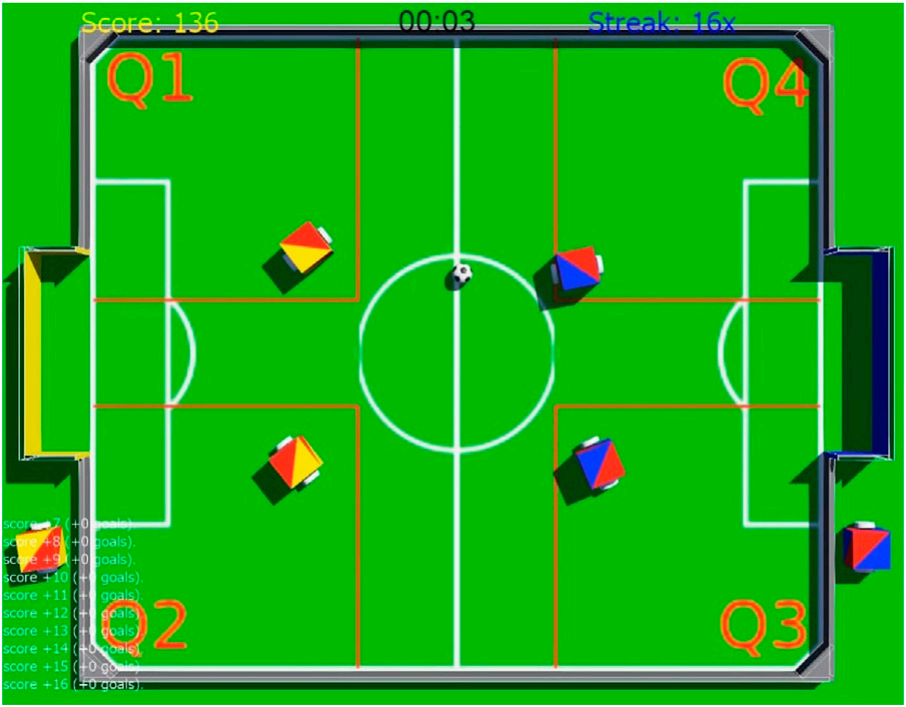
A screenshot of adjusted field for technical challenge in which robots (all of them programmed by one team) had to pass the ball between four field sections (starting from Q1, *via* Q2, Q3, Q4 and back to Q1) without leaving red squares.

Each of the challenges was evaluated by an automatic referee that was developed specifically for this purpose. In fact, each challenge had its own dedicated environment that allowed the implementation of extra features. Challenge number one didn’t require the addition of special features, since it consisted only of a regular robot and the ball in the field. In challenge number two, the robots’ goal was to pass the ball between each other. In this challenge, all four robots in the field illustrated in Figure 4 are programmed by the same team. They have to pass the ball between the four field sections indicated by the red squares, from Q1 to Q2, then to Q3, to Q4, and back to Q1. The robots are not allowed to leave their original red square. Finally, challenge number three requires the use of a new sensor, namely a *Lidar*. To be able to participate on this challenge, students were required to investigate and learn how to use the simulated *Lidar* sensor provided by Webots^17^. For each of the challenges the organizers awarded the best teams based on their overall score.

#### 3.4.6 Interviews

**FIGURE 5:**
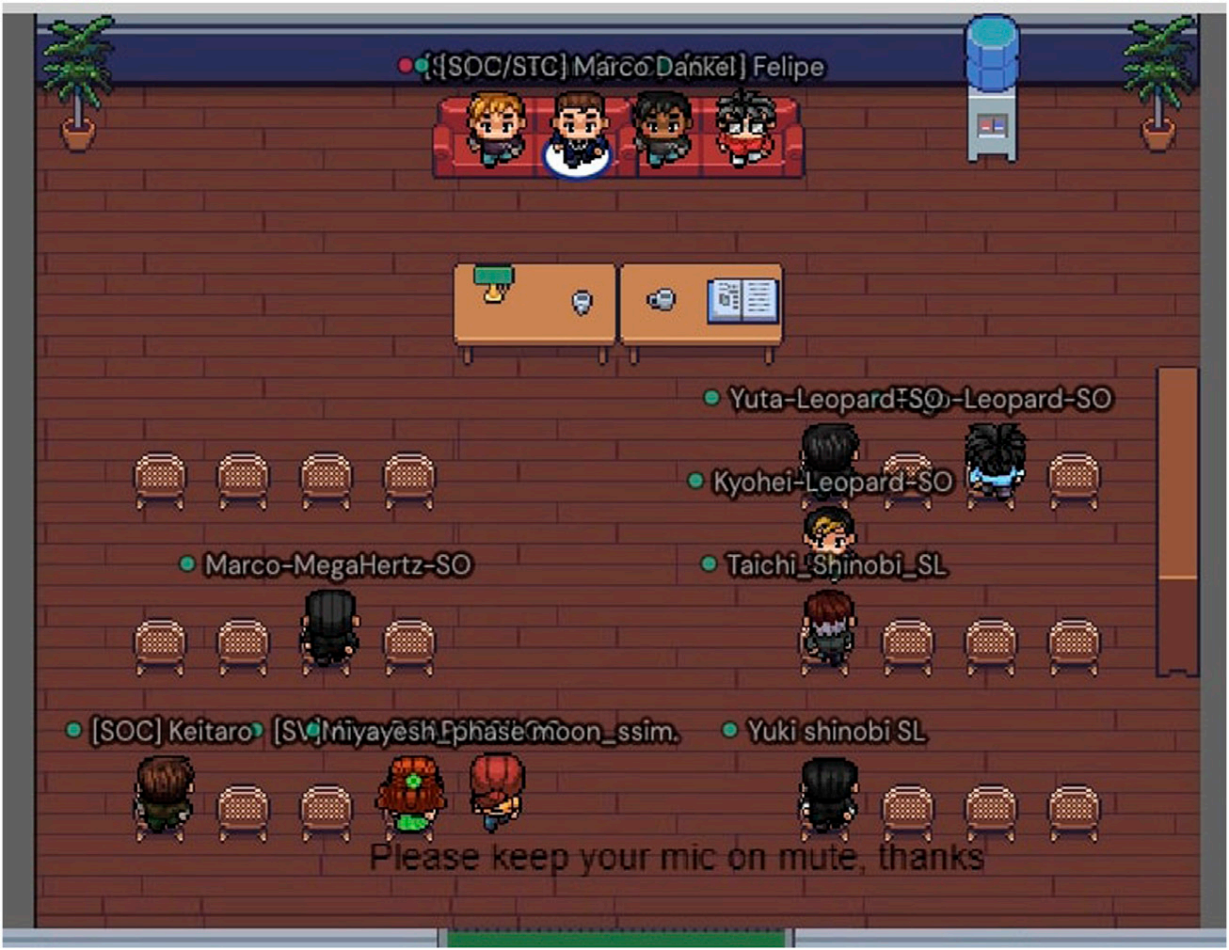
A screenshot of a sample GatherTown interview room.

As the aim of RoboCupJunior is to help technically-inclined students grow into well-rounded STEM professionals, one of the standard parts of the in-person competitions are so called “interviews” in which the team presents their work to a panel of judges. One of the limitations of organizing them during in-person competitions was the inability to make them open and letting anyone hear the team’s presentation—this was generally not possible due to physical space limitations. In the virtual setup, however, this limitation ceased to be a problem and the interviews were held in an open fashion with anyone invited to join. A sample GatherTown interview room can be seen in Figure 5.

Note that the interviews were primarily used for discussion on the team’s solutions of the Technical Challenges described above. The programs they submitted for the SoccerSim Individual competitions were the topic of the Poster session.

#### 3.4.7 Poster session

**FIGURE 6:**
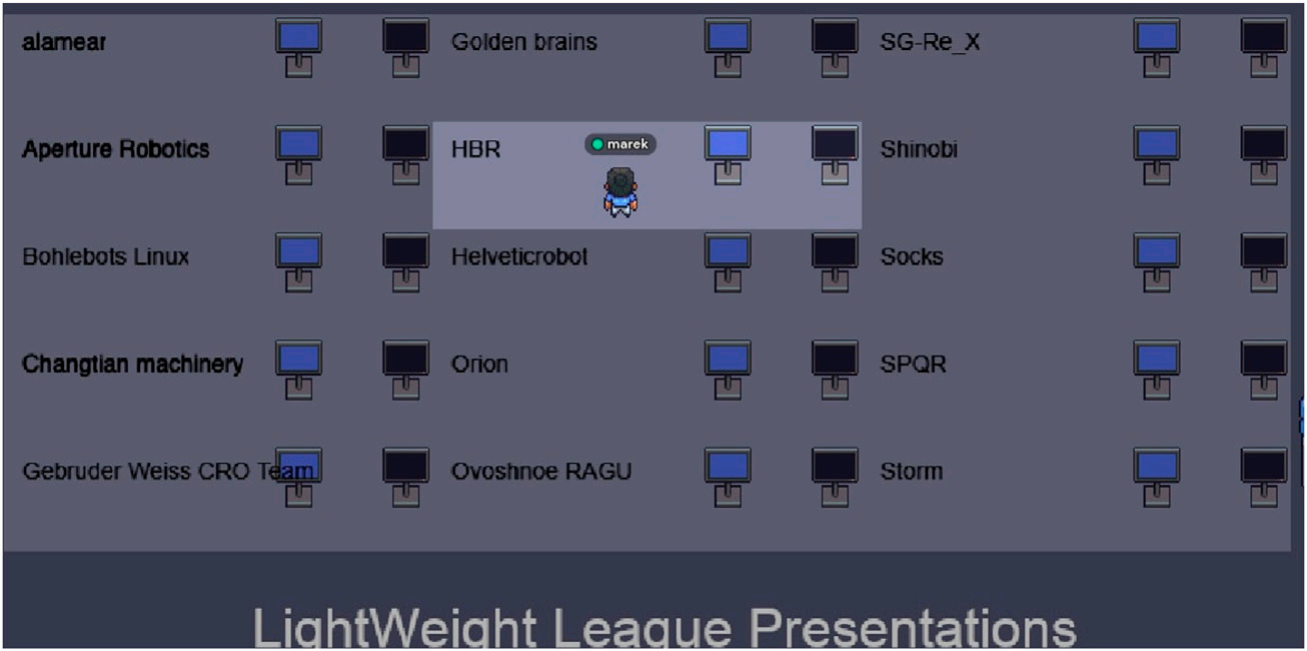
A screenshot of a poster area in GatherTown.

One of the fundamental ideas the RoboCup and RoboCupJunior competitions are built on, is the idea of learning and sharing knowledge. In order to provide a safe avenue for doing so, and also to provide the participants a glimpse of what scientific communication looks like, a poster session has been organized. During this session, a poster stand has been created for each team and a timeslot during which each team needed to have at least a single team member available to discuss their team’s poster in greater detail. This provided the teams a way of learning about the other teams’ approaches to the common problems and gave the opportunity for impromptu technical discussions. An image of a poster session area in GatherTown can be seen in Figure 6.

#### 3.4.8 Live stream of SoccerSim matches

In order to make the SoccerSim Individual competition a bit more dramatic and at the same time closer to professional soccer games, we organized a commented live stream for selected Quarter finals and Finals matches. These were again organized *via* GatherTown, with the commentators being present directly within its interface. Since the results of the live streamed matches were not known beforehand, they generally amassed considerable interest and also seemed to serve an important social function during a virtual event.

### 3.5 Participation numbers

In this section we present a comparison between the number of participants in the Soccer Simulation competitions and previous RoboCupJunior Soccer competitions. Table 2 presents a summary of the number of participants in recent years. The table shows the data for the Soccer Simulation competition at the RoboCup Worldwide 2021 (SoccerSim @RC Worldwide 2021), the Soccer Simulation Demo from February 2021 (SoccerSim Demo 2021), and the virtual poster presentations of RCJ Soccer 2020 (Soccer Virtual Poster 2020). The number of participants at the physical RCJ Soccer competition of 2019 (RCJ Soccer 2019) is also presented as a reference.

**TABLE 2:** Summary of the number of participant teams and countries in competitions of RoboCupJunior Soccer during the past three virtual events (in 2020 and 2021) as well as a physical event (in 2019).

	SoccerSim @RC Worldwide 2021	SoccerSim demo 2021	Soccer virtual poster 2020	RCJ soccer 2019
Registered teams	61	50	20	60
Actually participated	39	41	20	53
Number of countries	17	18	9	23

It is interesting to notice the difference in the number of participants between the years 2020 and 2021, both during the pandemic restrictions. In the first year of the pandemic, we were expecting that more teams would want to participate in some sort of event related to the RoboCup Junior. Both the Virtual Poster session of 2020 and the SoccerSim Demo competition of 2021 were free of charge, but more than twice as many teams participated in the simulation competition when compared to the virtual poster presentations. This indicates that students are much more motivated by the actual competition environment. The number of registered teams for the official SoccerSim competition at RoboCup Worldwide 2021 was even higher than the number of teams registered for the demo competition. However, fewer teams actually participated, probably due to the fact that the official competition is not a free event. Some teams also mentioned in their post-event feedback that it was more difficult for them to participate in a virtual event rather than a physical one, as they were not absolved from their other school duties during the virtual event, which would not be the case when they would travel to a physical event.

We also note that while the number of registered teams was higher for SoccerSim @RC Worldwide 2021 (60 as opposed to 61), many more teams eventually participated in the physical RCJ Soccer 2019 event (52 as opposed to 39) and these teams also came from more countries (23 as opposed to 17). The reason why this is the case has to do with the mechanics of the organization for the physical event, where the organization committee aims to invite the champions of specific countries, in proportion to the size of their regional events, with each country getting at least one spot. This is in direct contrast with the organization of SoccerSim @RC Worldwide 2021, where all the interested teams have been invited, in an attempt to give a wide variety of teams a chance to participate, as the size constraints of the physical venue did not apply in this case. Except for a single exception, however, the teams at SoccerSim @RC Worldwide 2021 came from the same countries as in RCJ Soccer 2019. During Soccer Virtual Poster 2020 the registration was directly tied with the actual poster submission, which explains why the same number of teams registered and actually participated. We would also like to emphasize that the numbers of registered and actually participating teams are difficult to compare directly, as RCJ Soccer has a 20 years tradition whereas SoccerSim @RC Worldwide 2021 has been a completely new event.

### 3.6 Participants’ experience at RoboCup Worldwide 2021

After the RoboCup Worldwide 2021 event, we sent out a survey to all teams that participated in the Junior Soccer competitions. Out of the roughly 60 teams that registered for the competition, about 34 have shared some feedback.

The first two questions focused on the participant’s experience with the SoccerSim @RC Worldwide 2021 competition. The average score obtained for the question “On the scale of 1–10, how much did you like this year’s Soccer @RC Worldwide 2021 competition?” was 8.11 whereas the median value was 8.5. On the next question which asked “On the scale of 1–10, how likely are you to suggest RCJ Soccer to a friend or a colleague?” we obtained an average of 8.85 with the median value of 10. This indicates that despite the new format, the participants generally found the event to be a positive experience which they would be happy to share with their friends and others.

The Soccer Simulation teams were also specifically asked: “Which part of the competition should we try to improve for the future?” Students could select more than one option and add free comments. Their answers were distributed as follows:

• 53%—Live Stream of SoccerSim matches
• 53%—SoccerSim SuperTeam competition
• 33%—Information about the competition/daily gatherings
• 26%—Interviews
• 20%—Poster session

As it can be seen, according to more than half of the participants, the most important items to be improved are the live stream of SoccerSim matches and the SoccerSim SuperTeam competition. All matches could be watched by the students using the visualization tool depicted in Figure 3, but only after they already knew about the results. Only a selected number of quarter finals and the final matches had a commented live stream. Results of those matches were, of course, unknown to the public and the teams. The livestream is, indeed, an important point of improvement for the next competition. About the SuperTeam competition, we heard from students that it is very difficult to collaborate with another team on-line, especially if there is a great time-zone distance. We will also address this issue for the next competition.

Perhaps the most important question to detect students’ satisfaction was: “If we kept the SoccerSim challenge running in the future, would you participate?” From those who participated in the SoccerSim competition, 80% answered YES, and 20% answered MAYBE. There was no negative answer, which illustrates that students are enthusiastic about the soccer simulation challenge.

## 4 Discussion

The COVID-19 pandemic has undoubtedly posed a significant challenge for many organizers of robotics events, especially competitions, with RoboCupJunior being no exception. Despite the obvious organizational difficulties, it also helped spearhead the adoption of simulated environments.

As we discussed in Section 2, the simulated environment developed for RoboCupJunior Soccer needed to fit a few criteria, such as an open-source license, open contribution model and support for the Python programming language. SoccerSim, the simulated environment that the Technical Committee (with the help of multiple students, volunteers and other contributors) has developed does indeed fit all of these criteria. It is based on the Webots simulator, which is licensed under the terms of the Apache 2 open-source license. The SoccerSim code itself is also licensed under the terms of the Apache 2 license. The Webots framework supports the Python programming language, which is also the language in which SoccerSim is implemented. The simulated robots can be programmed in multiple programming languages but SoccerSim specifically supports Python and even contains Python-based sample code that allows the students to quickly start experimenting with their own robots. SoccerSim is also distributed with a step-by-step quick start guide which goes over all the functionality SoccerSim provides from the programmer’s point of view.

The development of SoccerSim, as well as the rules, has profited immensely from direct interaction with and feedback from the community. As noted in Section 3.3, various bugs that were previously left unnoticed ended up being fixed thanks to this involvement. Furthermore, as Section 3.2 describes, organizing the Demo competition was critical to confirm the need for a specific submission system, while also providing evidence that the Webots-based simulator is an attractive choice for the RoboCupJunior’s target audience of mostly high school students. The competition has been run in an “open by default” mode, which meant that all the code submissions to the Demo competition were released at its conclusion. This meant that all the participants could learn and take inspiration from the winning submissions.

Finally, based on the feedback we received before, during and after the conclusion of the RoboCup Worldwide 2021 competition, we conclude that SoccerSim as the simulated environment, along with the activities outlined in Section 3.4, can be considered a viable alternative to a physical competition focused on robot soccer. Thanks to the significantly lower initial investment (the only requirement is an affordable computer as opposed to a robotics platform), the SoccerSim challenge at RoboCup Worldwide 2021 hosted teams that do not normally participate in RoboCupJunior Soccer—even from nations where RoboCupJunior community does not exist yet. We also believe that the combination of Webots (an easily installable, full fledged IDE) with Python (a beginner friendly programming language) resulted in an attractive challenge with a very low barrier to entry. As one of the participants wrote in their feedback, “we learned Python just to participate in RoboCupJunior SoccerSim!”.

### 4.1 Current status of SoccerSim

**FIGURE 7:**
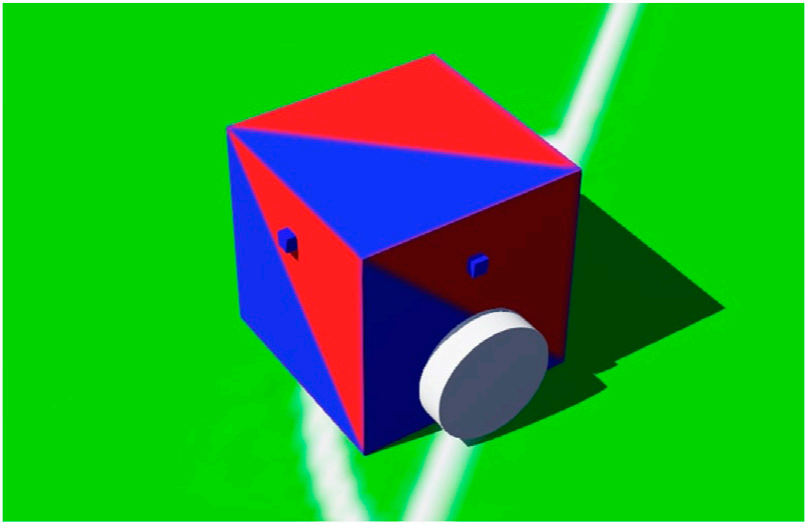
Detailed view on two out of four ultrasonic sensors mounted on sides of the robot (small blue cubes).

Based on the feedback from participants, team mentors, technical and organizing committees, we decided to further develop and improve the SoccerSim. The general idea was to approach physical robots in terms of sensors, eventually reaching parity with the physical RoboCupJunior Soccer competition. Receiving ground-truth data on vital attributes of the simulated environment like ball and robot position at all times is not very realistic. Instead, we put infra-red emitter into the ball, which emits the signal to objects located within a nearby radius. The robots are actually forced to make use of the communication because the signal of the ball is not visible everywhere due to its limited range and the possibility of other robots blocking it. Another improvement we made is in the area of positioning. Every robot is capable of reading its exact position using the *GPS* sensor. The rotation of the robot can be read using the *Compass* sensor. The returned vector indicates the north direction in the coordinate system of the Compass device. Last but not least, there are four *ultrasonic* sensors mounted on every robot (each side of the robot having one, see Figure 7). Since the exact position of the robot may be retrieved from GPS, these sensors are useful for detecting the opponent’s robots. All sensor data has some noise to make it more realistic.

In terms of codebase quality, the SoccerSim’s repository features a Continuous Integration (CI) pipeline, which ensures conformity to a code style. Moreover, it also ensures the referee’s code is covered by unit-tests to ensure its stability. Apart from increasing SoccerSim’s robustnes, these changes also make it easy for outside contributors to rest assured that their suggested changes do not affect the existing functionality.

### 4.2 Future work

Even though the software is stable based on our testing, we would like to enhance it by making it even closer to the real world’s RoboCupJunior Soccer competition. In fact, Webots supports Camera device^18^ to be attached to the robot while computing OpenGL rendered images and later using it for doing object recognition or segmentation. This is especially challenging because it requires graphic card for computing, otherwise the simulation will be quite slow. Thanks to the powerful IDE Webots has, we would like to allow participants design their own robots based on some guidelines. It would be then not only competition for programmers, but also designers.

The ultimate goal is to develop a system for organizing the whole tournament, from accepting teams’ submissions, generating and simulating the tournament’s fixtures, to displaying results and videos at a single place. Provided all of these steps are automated, this would allow us to run something like a “SoccerSim league” much more often (say each month) than just once a year, similarly to various other “bot” competitions. Seeing the positive response to the SoccerSim Demo and Worldwide tournaments, we believe this could help foster a community of students interested in artificial intelligence and robotics, and being a nice complement to the already existing RoboCupJunior challenges.

## Data Availability

The datasets generated for this study are available on request to the corresponding authors.

## Footnotes

1
https://junior.robocup.org/rescue-simulation-league-former-cospace-league/

2
https://processing.org/

3
https://www.gather.town/

4
https://www.tiobe.com/tiobe-index/python/

5
https://junior.forum.robocup.org/

6
https://junior.forum.robocup.org/t/soccer-simulation-demo-competition-2021/1709

7
https://github.com/RoboCupJuniorTC/rcj-soccersim

8
https://jsonlines.org/

9
https://github.com/RoboCupJuniorTC/rcj-soccersim/

10
https://junior.forum.robocup.org/

11
https://github.com/RoboCupJuniorTC/soccersim-checker

12
https://junior.forum.robocup.org/t/soccer-simulation-demo-2021-issues-and-questions/1766

13
https://junior.forum.robocup.org/t/call-for-suggestions-technical-challenges/2072/2

14 Note that the programs did not change mid-tournament—the last submitted version of each team has been used for simulation of all matches.

15
https://robocupjuniortc.github.io/soccer-2021/worldcup-finals.html

16
http://junior.robocup.org/wp-content/uploads/2018Rules/soccer_rules_final_2018.pdf

17
https://cyberbotics.com/doc/reference/lidar?tab-language=python

18
https://cyberbotics.com/doc/reference/camera
